# Association of Low Muscle Mass and Combined Low Muscle Mass and Visceral Obesity with Low Cardiorespiratory Fitness

**DOI:** 10.1371/journal.pone.0100118

**Published:** 2014-06-17

**Authors:** Tae Nyun Kim, Man Sik Park, You Jeong Kim, Eun Ju Lee, Mi-Kyung Kim, Jung Min Kim, Kyung Soo Ko, Byoung Doo Rhee, Jong Chul Won

**Affiliations:** 1 Department of Internal Medicine, Inje University College of Medicine, Busan, Korea; 2 Division of Endocrinology and Metabolism, Cardiovascular and Metabolic Disease Center, Inje University, Busan, Korea; 3 Department of Statistics, College of Natural Sciences, Sungshin Women’s University, Seoul, Korea; INIA, Spain

## Abstract

**Objective:**

Previous studies have shown that low cardiorespiratory fitness (CRF), visceral obesity and low muscle mass may share pathophysiological mechanisms, such as insulin resistance and chronic inflammation. In this study, we investigated whether low CRF is associated with low muscle mass, visceral obesity, and visceral obesity combined with low muscle mass.

**Research Design and Methods:**

The associations between CRF and low muscle mass and combined low muscle mass and visceral obesity were examined in 298 apparently healthy adults aged 20–70 years. Low muscle mass was defined using a skeletal muscle mass index (SMI) that was calculated using dual energy X-ray absorptiometry. Visceral obesity was defined as a visceral fat area (VFA) exceeding 100 cm^2^ in women and 130 cm^2^ in men. We classified the participants into 4 low muscle mass/visceral obesity groups according to SMI and VFA. CRF was measured using a cycle ergometer test.

**Results:**

CRF level correlated positively with SMI and negatively with VFA. Individuals with low muscle mass had lower CRF values than those without low muscle mass. After adjustment for age, sex, lifestyle factors, and markers for insulin resistance and inflammation, participants in the lowest quartile of CRF had an odds ratio (OR) for low muscle mass of 4.98 compared with those in the highest quartile (95% confidence interval (CI) = 1.19–12.99; *P* for trend = 0.001) and an OR for combined low muscle mass and visceral obesity of 31.46 (95% CI = 4.31–229.68; *P* for trend = 0.001).

**Conclusions:**

Individuals with lower CRF exhibited increased risk of low muscle mass and combined low muscle mass and visceral obesity. These results suggest that low CRF may be a potential indicator for low muscle mass and combined low muscle mass and visceral obesity in Korean adults.

## Introduction

It is well established that low cardiorespiratory fitness (CRF) is associated with a higher risk of developing cardiovascular disease (CVD) and mortality in asymptomatic individuals [1], [2]. Regular physical activity improves level of CRF and plays a role in both primary and secondary prevention of CVD [3], [4]. Therefore, a low CRF is regarded as one of the most modifiable CVD risk factors. Maximal aerobic capacity (Vo_2max_) was the objective CRF measure obtained in previous studies; Vo_2max_is a health-related component of physical fitness that relates to the ability of the circulatory and respiratory systems to supply oxygen during sustained physical activity [5]. Previous studies showed that Vo_2max_ was positively associated with muscle mass [6], [7], whereas it was suggested that body fat accumulation was negatively associated with maximal whole body oxygen uptake [8].

Although the etiology of combined low muscle mass and visceral obesity is still poorly understood, several proposed underlying mechanisms including aging, sedentary life style, insulin resistance and inflammation may be related to low CRF. Insulin resistance may be a strong contributor to low CRF, low muscle mass and combined low muscle mass and visceral obesity. In other words, low CRF is now regarded as a cause and consequence of insulin resistance [9]. In addition to insulin resistance, low CRF and combined low muscle mass and visceral obesity seem to have similar pathophysiological backgrounds, such as chronic inflammation, oxidative stress, and decreased physical activity. Kullo et al. reported that higher circulating levels of IL-6, C-reactive protein (CRP), and fibrinogen are independently associated with lower Vo_2max_ in asymptomatic men [10]. Furthermore, it is presumed that decreased physical activity, induced by low muscle mass, can cause a reduction of energy expenditure, insulin resistance and inflammation, which may result in visceral obesity and reduce the level of CRF in patients with low muscle mass. However, there is a paucity of research on the association of CRF with low muscle mass and combined low muscle mass and visceral obesity.

In the present study, we examined the association of low CRF with low muscle mass and combined low muscle mass and visceral obesity in a Korean population. For this purpose, we compared cardiometabolic parameters that are well known to be associated with low CRF between individuals with and without low muscle mass. Additionally, risk of low muscle mass and combined low muscle mass and visceral obesity by quartiles of Vo_2max_ were calculated after adjusting for potential confounding factors including markers of insulin resistance and inflammation.

## Research Design and Methods

### Subjects and Ethics Statement

We analyzed baseline data from across-sectional study to explore the association of adiposity and/or adipokine related to insulin resistance and inflammation and to evaluate their effects on combined low muscle mass and visceral obesity and health outcome [11]. The study cohort consisted of 298 adults aged ≥19 years (119 men and 179 women), who visited Ilsan Paik Hospital for a regular checkup during the period of March 2009 through August 2009. Eligible participants had no history of any type of diabetes, CVD (myocardial infarction, unstable angina, stroke or cardiovascular revascularization), stage 2 hypertension (resting blood pressure ≥160/100 mmHg), malignant disease, or severe renal or hepatic disease. Subjects taking medications that could affect body weight or body composition were excluded. Participants had their body composition analyzed by dual energy X-ray absorptiometry (DXA) and computed tomography (CT) to define low muscle mass and visceral obesity. Of the 298 subjects, 67 (26 men and 41 women between the ages of 19 and 30 years) were classified as a young healthy reference group. All participants provided written informed consent, and the Inje University Ilsan Paik Hospital Institutional Review Board approved the study protocol in accordance with the Declaration of Helsinki by the World Medical Association.

### Clinical and Laboratory Measurements

All blood samples were obtained in the morning after a 12-hour overnight fast and were immediately stored at −70°C for subsequent assays. Serum IL-6 and tumor necrosis factor-alpha (TNF-α) concentrations were also measured using ELISA (R&D Systems, Abingdon, UK). Fasting plasma glucose, serum triglycerides, total cholesterol, and high-density lipoprotein (HDL)-cholesterol levels were determined enzymatically using a chemistry analyzer (ADVIA 1650, Bayer, Ltd., Tokyo, Japan). High-sensitivity C-reactive protein (hsCRP) was measured by EIA (Modular P800; Roche, Basel, Switzerland). Plasma levels of insulin and leptin were measured by radioimmunoassay (Hitachi E170; Hitachi Ltd., Tokyo, Japan and Linco Research, St. Charles, MO, USA, respectively). The insulin resistance (IR) index was calculated from fasting plasma insulin, and plasma glucose levels were estimated by the homeostasis model assessment (HOMA) where HOMA = fasting plasma insulin (IU/mL) × fasting plasma glucose (mmol/L)/22.5. Metabolic syndrome was defined according to the criteria established by the National Cholesterol Education Program Adult Treatment Panel III using the adjusted waist circumference for Asians [12], [13].

### Assessment of Body Composition

Body mass index was calculated as weight/height^2^ (kg/m^2^), and waist circumference was measured at the midpoint between the lower border of the rib cage and the iliac crest. A whole body DXA scan was performed for each patient to measure total and regional lean mass (kg), total body fat (kg) and total body fat percentage (%) using fan-beam technology (QDR 4500; Hologic, Bedford, MA, USA). Appendicular skeletal muscle mass (kg) and skeletal muscle mass index (SMI [%] = total skeletal muscle mass [kg]/weight [kg]×100) were obtained as previously described [14], [15].

The abdominal adipose tissue area was quantified by CT (Somatom Plus 4; Siemens, Forchheim, Germany). Visceral fat area was calculated from a 10-mm CT slice scan image between the fourth and fifth lumbar vertebrae that was obtained during suspended respiration. VFA was quantified by delineating the intra-abdominal cavity at the internal aspect of the abdominal and oblique muscle walls surrounding the cavity and the posterior aspect of the vertebral body. Fat attenuation was determined by measuring the mean value of the pixels within a range of −190 to −30 Hounsfield units.

### Cardiorespiratory Fitness

Vo_2max_ is generally accepted as the criterion measure of CRF. CRF was obtained as previously described [16]. Vo_2max_ was ascertained by having the subjects exercise on a cycle ergometer (TKK3070 Active 10; Takei Co., Tokyo, Japan) for 13 minutes at gradually increasing intensity to a submaximal heart rate. During this time, the heartbeat was monitored, and built-in software calculated the anticipated Vo_2max_.

### Definitions of Low Muscle Mass, Visceral Obesity and Combined Low Muscle Mass and Visceral Obesity

Low muscle mass was defined as a SMI of 1 standard deviation (SD) below the sex-specific mean value for the young reference group [17], [18]. The cut-off point for low muscle mass was 36.3% in men and 28.5% in women. In this study, visceral obesity was defined as a VFA ≥100 cm^2^ in women and a VFA ≥130 cm^2^ in men; these are VFA levels are known to be highly associated with metabolic impairment. Combined low muscle mass and visceral obesity was defined as a high VFA combined with low relative skeletal muscle mass.

### Statistical Analysis

Baseline characteristics according to different combination of low muscle mass and visceral obesity are reported, and analyzed by gender. Numerical data are expressed as mean ± standard deviation (SD) or median (inter-quartile range). Differences in continuous variables between the four groups were evaluated using an analysis of variance (ANOVA) method or the Kruskal-Wallis H-test. The Turkey’s HSD method or the Wilcoxon rank-sum test as post-hoc test was also used to determine significant differences between the two groups. Spearman’s partial correlation analysis adjusting for age and gender was performed to determine the relationships between SMI, VFA, and CRF and other metabolic variables. We further divided the distribution of pooled CRF data into quartiles and used the logistic regression models to estimate odds ratios (ORs) and 95%confidence intervals (CIs), predicting low muscle mass and combined low muscle mass and visceral obesity using the highest quartile as the reference category. The logistic regression model was adjusted for age, sex, smoking status, alcohol consumption, systolic and diastolic blood pressure, total cholesterol, HDL-cholesterol, triglycerides, fasting plasma glucose, HOMA-IR, hsCRP, IL-6, TNF-α, and leptin. All statistical results based on 2-sided tests were obtained from SAS for Windows (version 9.20, SAS Institute Inc., Cary, NC, USA). A *P*-value <0.05 was considered statistically significant in all analyses.

## Results

### Characteristics of Study Subjects

The mean age of the participants considered in this study was 40.1±11.2 years, and 60.1% were women. No difference of age, hsCRP, leptin, systolic and diastolic blood pressure, and HOMA-IR was found between women and men. However, because there was a significant difference in BMI, WC, fasting plasma glucose, lipid profiles, IL-6, TNF-α, VFA, total skeletal muscle mass, SMI, and Vo_2max_ between women and men, subsequent statistical analyses were performed separately for women and men. The characteristics of the study population according to low muscle mass and visceral obesity groups are shown in Table 1 and 2. Subjects with neither low muscle mass nor visceral obesity (“normal group”) were younger than any other group in both men and women. Subjects with combined low muscle mass and visceral obesity had higher values of body mass index, waist circumference, VFA, systolic blood pressure, and diastolic blood pressure, serum triglycerides in both men and women than normal group. Regarding the markers for insulin resistance and chronic inflammation, there were significant differences in HOMA-IR, hsCRP and leptin levels between those with combined low muscle mass and visceral obesity and the normal group in both men and women. Furthermore, subjects with low muscle mass had lower CRF than those without low muscle mass in both men (31.4±6.2and36.7±6.3, respectively, P<0.001) and women (26.8±7.6 and 30.7±6.6, respectively, P<0.001). Moreover, CRF values were lower in the low muscle mass only (not visceral obese) and combined low muscle mass and visceral obesity group than in the normal group in both men and women.

**Table 1:** Clinical, anthropometric, and metabolic characteristics of female study subjects.

	Neither low muscle massnor visceral obesity	Low muscle mass	Visceral obesity	Low muscle mass andvisceral obesity	*P*-value
	(n = 78)	(n = 59)	(n = 18 )	(n = 24)	
Age (years)	34.6±10.1^a^	41.9±9.9^b^	45.7±9.5^b^	48.2±8.7^b^	<0.001
Body mass index (kg/m^2^)	21.8±2.2^a^	23.7±2.5^b^	25.4±2.3^c^	27.6±2.6^d^	<0.001
Waist circumference (cm)	75.9±5.9^a^	80.1±5.6^b^	84.6±6.2^c^	90.5±4.4^d^	<0.001
Systolic blood pressure (mmHg)	105.2±10.7^a^	105.2±10.7^a^	120.8±12.9^b^	122.4±17.3^b^	<0.001
Diastolic blood pressure (mmHg)	64.8±7.5^a^	66.7±6.2^a^	72.6±8.8^b^	76.1±10.8^b^	<0.001
Total cholesterol (mmol/L)	4.5±0.7	4.5±0.8	4.6±0.7	4.9±0.8	0.100
HDL cholesterol (mmol/L)	1.4±0.3^a^	1.2±0.3^ab^	1.1±0.3^b^	1.1±0.3^b^	<0.001
Triglycerides (mmol/L)	0.7 (0.6, 1.0)^a^	0.8 (0.6, 1.1)^ab^	1.1 (0.9, 1.2)^bc^	1.2 (1.0, 1.4)^c^	<0.001
Fasting plasma glucose (mmol/L)	4.8±0.4^a^	4.9±0.5^ab^	5.2±0.9^b^	5.3±1.5^b^	0.006
HOMA-IR	0.7 (0.4, 1.1)^a^	1.0 (0.7, 1.4)^b^	1.6 (1.3, 2.5)^c^	1.5 (1.0, 2.5)^c^	<0.001
hsCRP (mg/L)	0.0 (0.0, 0.1)^a^	0.0 (0.0, 0.1)^ab^	0.1 (0.0, 0.2)^bc^	0.1 (0.1, 0.1)^c^	<0.001
Interleukin-6 (pg/mL)	1.8 (1.4, 2.1)^a^	1.8 (1.6, 2.2)^ab^	1.8 (1.7, 2.1)^ab^	2.1 (1.8, 2.5)^b^	0.012
TNF-α(pg/mL)	7.3±1.2^ab^	6.9±1.3^b^	7.4±1.1^ab^	8.7±6.1^a^	0.033
Leptin (µg/L)	5.2 (3.6, 8.7)^a^	9.5 (6.1, 15.2)^bc^	10.7 (5.4, 16.2)^ac^	13.2 (9.1, 16.7)^c^	<0.001
Visceral fat area (cm^2^)	43 (29, 59)^a^	62 (50, 78)^b^	113 (110, 132)^c^	134 (115, 153)^c^	<0.001
Total skeletal muscle mass (kg)	17.4±2.8^ab^	15.9±1.7^c^	18.9±2.4^a^	17.1±2.0^bc^	<0.001
SMI (%)	31.0±2.3^a^	27.0±1.2^c^	29.8±1.0^b^	25.7±2.2^d^	<0.001
CRF (mL/kg/min)	31.6±6.5^a^	27.3±7.5^b^	27.1±6.0^b^	25.6±8.0^b^	<0.001

CRF, Cardiorespiratory fitness; HDL, high density lipoprotein; HOMA-IR, homeostasis model assessment of insulin resistance; hsCRP, high-sensitivity C-reactive protein; SMI, skeletal muscle index; M, male; F, female.

Data are presented as mean ± SD or Median (Inter-quartile range).

*P*-values were calculated from the one-way analysis of variance (ANOVA) or the Kruskal-Wallis H test.

a,b,c,d Same letter indicates no statistical difference based on Tukey’s HSD method or Wilcoxon rank-sum test.

**Table 2:** Clinical, anthropometric, and metabolic characteristics of male study subjects.

	Neither low muscle massnor visceral obesity	Low muscle mass	Visceral obesity	Low muscle mass andvisceral obesity	*P*-value
	(n = 55)	(n = 24)	(n = 9)	(n = 31)	
Age (years)	35.7±10.7^a^	40.5±10.7^ab^	42.6±8.4^ab^	47.4±10.9^b^	<0.001
Body mass index (kg/m^2^)	24.1±2.7^a^	24.7±3.1^a^	28.0±1.4^b^	27.5±2.5^b^	<0.001
Waist circumference (cm)	86.0±6.4^a^	89.6±6.8^ab^	94.0±3.1^bc^	97.6±7.0^c^	<0.001
Systolic blood pressure (mmHg)	117 (109, 131)^a^	119 (108, 128)^ab^	129 (128, 135)^ab^	128 (119, 142)^b^	0.004
Diastolic blood pressure (mmHg)	72.9±9.2^a^	74.7±8.0^ab^	81.3±5.5^b^	81.6±9.9^b^	<0.001
Total cholesterol (mmol/L)	4.8±0.7	4.9±0.7	5.2±0.9	4.7±0.9	0.314
HDL cholesterol (mmol/L)	1.1±0.3	1.2±0.3	1.0±0.2	1.1±0.3	0.328
Triglycerides (mmol/L)	1.3 (0.9, 1.7)^a^	1.1 (0.8, 1.6)^a^	2.2 (1.6, 3.6)^b^	1.9 (1.1, 2.8)^b^	<0.001
Fasting plasma glucose (mmol/L)	4.9 (4.7, 5.2)	5.0 (4.6, 5.4)	5.3 (5.1, 5.6)	5.2 (4.8, 5.6)	0.062
HOMA-IR	0.9 (0.6, 1.3)^a^	0.9 (0.6, 1.5)^ab^	2.0 (1.0, 2.9)^bc^	1.8 (1.2, 2.6)^c^	<0.001
hsCRP (mg/L)	0.0 (0.0, 0.1)^a^	0.1 (0.0, 0.1)^ab^	0.1 (0.0, 0.3)^b^	0.1 (0.0, 0.2)^b^	<0.001
Interleukin-6 (pg/mL)	1.5 (0.1, 1.9)^a^	2.0 (1.6, 2.4)^b^	1.7 (1.6, 2.1)^ab^	1.8 (0.3, 2.5)^ab^	0.014
TNF-α(pg/mL)	8.1±1.3	7.5±1.2	7.3±1.2	8.0±1.3	0.104
Leptin (µg/L)	2.5 (1.5, 3.5)^a^	3.3 (2.1, 3.9)^ab^	2.9 (2.4, 5.4)^ab^	4.8 (3.2, 6.8)^b^	<0.001
Visceral fat area (cm^2^)	73 (59, 103)^a^	103 (78, 119)^a^	147 (137, 157)^b^	158 (141, 206)^b^	<0.001
Total skeletal muscle mass (kg)	27.6±3.1^ab^	25.0±4.4^b^	30.2±2.8^a^	27.3±3.7^ab^	0.002
SMI (%)	39.0±2.1^a^	34.6±1.9^c^	37.2±0.5^b^	33.9±1.8^c^	<0.001
CRF (mL/kg/min)	37.4±5.8^a^	32.1±5.7^b^	32.3±7.7^b^	30.9±6.7^b^	<0.001

CRF, Cardiorespiratory fitness; HDL, high density lipoprotein; HOMA-IR, homeostasis model assessment of insulin resistance; hsCRP, high-sensitivity C-reactive protein; SMI, skeletal muscle index.

Data are presented as mean ± SD or Median (Inter-quartile range).

*P*-values were calculated from the one-way analysis of variance (ANOVA) or the Kruskal-Wallis H test.

a,b,c,d Same letter indicates no statistical difference based on Tukey’s HSD method or Wilcoxon rank-sum test.

### Correlation of SMI, VFA and CRF with Cardiometabolic Risk Factors

Table 3 shows the correlation analysis of SMI, VFA, and CRF with other major metabolic variables in the study population. After adjusting for age and gender, SMI and CRF tended to be negatively correlated with most of the markers for insulin resistance and chronic inflammation, including HOMA-IR, hsCRP, IL-6, leptin, and the number of metabolic syndrome components. In contrast, VFA was positively associated with the cardiometabolic risk factors, whereas CRF and HDL-cholesterol were negatively correlated with VFA. Additionally, CRF was positively correlated with total skeletal muscle mass and SMI.

**Table 3:** Spearman’s partial correlation analysis of skeletal muscle index (SMI), visceral fat area (VFA) and cardiorespiratory fitness (CRF) with cardiometabolic parameters adjusting for age and gender effects.

	SMI		VFA		CRF	
	*r*-value	*P*-value	*r*-value	*P*-value	*r*-value	*P*-value
Systolic blood pressure	−0.106	0.084	0.403	<0.001	−0.219	<0.001
Diastolic blood pressure	−0.107	0.080	0.390	<0.001	−0.230	<0.001
Total cholesterol	0.017	0.783	0.035	0.574	−0.017	0.777
Triglyceride	−0.090	0.142	0.374	<0.001	−0.136	0.026
HDL-cholesterol	0.192	0.002	−0.356	<.001	0.177	0.004
Glucose	−0.040	0.513	0.151	0.013	0.028	0.652
HOMA-IR	−0.251	<0.001	0.526	<0.001	−0.255	<0.001
Number of MetS components	−0.269	<0.001	0.573	<0.001	−0.256	<0.001
hsCRP	−0.231	<0.001	0.450	<0.001	−0.256	<0.001
Interleukin-6	−0.089	0.148	0.117	0.056	−0.163	0.008
TNF-α	0.012	0.840	0.099	0.107	−0.041	0.502
Leptin	−0.496	<0.001	0.463	<0.001	−0.312	<0.001
Visceral fat area	−0.311	<0.001			−0.339	<0.001
SMI			−0.311	<0.001	0.317	<0.001

HDL, high density lipoprotein; HOMA-IR, homeostasis model assessment insulin resistance; hsCRP, high sensitivity C-reactive protein; MetS, metabolic syndrome; SMI, skeletal muscle mass index TNF-α, tumor necrosis factor-α.

*r*-value stands for the Spearman partial correlation coefficient.

### Multiple Logistic Regression Analysis for Low Muscle Mass and Combined Low Muscle Mass and Visceral Obesity

Multiple logistic regression analyses were performed with low muscle mass and combined low muscle mass and visceral obesity as a dependent variable. The ORs and 95% CIs calculated for each quartile of CRF was shown in Table 4. In an unadjusted model, subjects in the third quartile for CRF value (OR = 3.42, 95% CI = 1.30–8.96), the second quartile (OR = 4.03, 95% CI = 1.56–10.40) and the first quartile (OR = 5.88, 95% CI = 2.33–14.84) had a significantly higher risk for low muscle mass compared to those in the fourth quartile (*P* for trend <0.001). Even with further adjustment for potential confounding factors, including insulin resistance and inflammation, the associations remained statistically significant (*P* for trend = 0.001). In addition, visceral obesity was associated with low CRF after controlling for all covariates. Furthermore, for combined low muscle mass and visceral obesity, subjects in the third quartile for CRF value (OR = 7.63, 95% CI = 1.18–49.47), the second quartile (OR = 7.50, 95% CI = 1.19–47.45), and the lowest (OR = 31.46, 95% CI = 4.31–229.68) had significantly higher risk for combined low muscle mass and visceral obesity controlling for all covariates than those in the highest quartile (*P* for trend = 0.001).

**Table 4:** Unadjusted and adjusted odds ratios (ORs) with 95% confidence intervals (CIs) of having low muscle mass only, visceral obesity only and combined low muscle mass and visceral obesity by quartiles of CRF (mL/kg/min) adjusting for potential compounding factors.

	Quartiles of CRF (mL/kg/min)					
Low muscle mass	Q4 (highest)	Q3	Q2	Q1 (lowest)		*P* for trend
Unadjusted	1	1.55 (0.79, 3.07)	2.47 (1.26, 4.85)	4.82 (2.40, 9.68)		<0.001
Model 1	1	1.63 (0.80, 3.33)	2.58 (1.25, 5.33)	4.58 (2.09, 10.02)		<0.001
Model 2	1	2.03 (0.93, 4.45)	2.90 (1.30, 6.45)	5.55 (2.35, 13.12)		<0.001
Model 3	1	1.80 (0.75, 4.35)	2.06 (0.86, 4.96)	4.98 (1.91, 12.99)		0.001
**Visceral Obesity**						
Unadjusted	1	1.87 (0.81, 4.30)	2.74 (1.23, 6.11)	3.70 (1.67, 8.21)		0.001
Model 1	1	2.12 (0.86, 5.22)	3.49 (1.42, 8.56)	4.60 (1.80, 11.74)		0.001
Model 2	1	2.08 (0.68, 6.40)	4.18 (1.43, 12.24)	5.36 (1.69, 17.01)		0.002
Model 3	1	2.10 (0.52, 8.40)	4.26 (1.11, 16.41)	6.48 (1.56, 26.89)		0.004
**Combined low muscle mass and visceral obesity**						
Unadjusted	1	2.07 (0.78, 5.53)	2.23 (0.84, 5.89)	3.74 (1.47, 9.50)		0.006
Model 1	1	2.36 (0.82, 6.80)	2.99 (1.02, 8.74)	5.99 (1.97, 18.18)		0.002
Model 2	1	3.60 (0.97, 13.33)	4.69 (1.29, 16.98)	10.54 (2.66 41.73)		0.001
Model 3	1	7.63 (1.18, 49.47)	7.50 (1.19, 47.45)	31.46 (4.31, 229.68)		0.001

Model 1: adjusted for age and sex.

Model 2: adjusted for age, sex, smoking status, and alcohol consumption, systolic and diastolic blood pressure, total cholesterol, HDL-cholesterol, triglycerides, fasting plasma glucose.

Model 3: adjusted for age, sex, smoking status, and alcohol consumption, systolic and diastolic blood pressure, total cholesterol, HDL-cholesterol, triglycerides, fasting plasma glucose, HOMA-IR, hsCRP, interleukin-6, TNF-α, and leptin.

## Discussion

We found a strong inverse association between CRF levels and low muscle mass and combined low muscle mass and visceral obesity in Korean adults. This association was independent of age, sex, lifestyle factors, and markers of insulin resistance and inflammation. Our data suggest that low CRF was associated with an increased risk of having low muscle mass and combined low muscle mass and visceral obesity in Korean adults.

A review of the literature reveals that low CRF is associated with visceral adipose tissue accumulation and risk of developing metabolic syndrome and CVD in adults, and that low CRF is a significant mortality predictor in older adults [19], [20]. Skeletal muscle loss plays an important role in the age-associated loss of CRF [7]. de Oliveira et al. reported that fat-free mass and quadriceps strength are significantly related to CRF in older women, and that women with low muscle mass have lower levels of CRF than those without low muscle mass [6]. Ryu et al. showed that intense physical activity (as self-reported on the International Physical Activity Questionnaire) is associated with reduced risk of low muscle mass and combined low muscle mass and obesity in older Korean adults [21]. However, visceral obesity, which is known to be most closely associated with metabolic deterioration and catabolic cytokine, was not measured in that study. Furthermore, they did not perform cardiorespiratory exercise testing, which is considered the gold standard in determining exercise capacity. Interestingly, the present study showed that each group with low muscle mass(low muscle mass only group and combined low muscle mass and visceral obesity group) had a lower Vo_2max_ compared to the normal group even in young and middle aged adults.

The proposed mechanisms involved in both low muscle mass and visceral obesity include physical inactivity, insulin resistance, chronic inflammation, and changes in hormonal milieu [22]. The decreases in physical activity may reduce muscle mass and strength as well as trigger visceral fat accumulation. The influence of insulin resistance on low muscle mass and combined low muscle mass and visceral obesity has recently been explored. Using data from the National Health and Nutrition Examination Survey III, Srikanthan et al. showed that low muscle mass exacerbates obesity-associated insulin resistance [23]. Low levels of CRF are also closely associated with higher insulin resistance in adults [24]. Furthermore, insulin resistance may enhance muscle catabolism because insulin acts as a powerful signal for proteins anabolism [25]. Treatment aimed at improving insulin resistance, such as weight loss and exercise training, have shown some benefit in patients with low CRF and combined low muscle mass and visceral obesity [26], [27]. The present study revealed an increased insulin resistance index in subjects with low muscle mass compared to those without low muscle mass. In addition, SMI tended to be negatively correlated with insulin resistance. A significant relationship between CRF (which may be protective against visceral fat accumulation) and insulin resistance was also found.

Systemic low-grade inflammation has been recognized as an important causative factor for low muscle mass as well as combined low muscle mass and visceral obesity [22], [28]. Cross-sectional and longitudinal studies support the hypothesis that there is an association between inflammation and low muscle mass [29]. TNF-α and IL-6 are the most reported inflammatory markers [29]. Cesari et al. reported that CRP and IL-6 are negatively associated with fat-adjusted appendicular lean mass and positively associated with total fat mass [30]. In this study, individuals with low muscle mass had increased levels of hsCRP and leptin compared to those without low muscle mass. Moreover, serum hsCRP and leptin concentrations were closely and negatively correlated with both SMI and CRF (*P*<0.001, respectively), which suggests that inflammation may be an important underlying factor associated with both low muscle mass and low CRF.

In addition to insulin resistance and inflammation, CRF was negatively associated with VFA in our study, but was positively related to muscle mass index such as SMI and total skeletal muscle mass. The metabolic effects of low muscle mass including a decrease in resting metabolic rate and reduction in physical activity, may lead to an increase of fat mass, particularly visceral fat. Visceral obesity directly affects inflammation and insulin resistance and may cause functional limitation, which in turn negatively affects skeletal muscle mass, contributing to the development and progression of combined low muscle mass and visceral obesity [31]. Therefore, low muscle mass and obesity may potentiate each other and have a synergistic effect on physical capacity, chronic metabolic disorders and CVD. The present study exhibited that low muscle mass as well as combined low muscle mass and visceral obesity are independently associated with low CRF, which is strongly associated with higher risk of all-cause and cardiovascular disease mortality.

Decreased physical activity induces loss of skeletal muscle mass as well as visceral fat accumulation through reduced energy expenditure. However, progressive resistance exercise can improve physical function and may counteract low muscle mass [32]. Davidson et al. randomized 135 sedentary, abdominally obese men and women into 4 groups: resistance exercise, aerobic exercise, a combination of resistance and aerobic exercise (combined exercise), and a non-exercise control group [33]. Insulin resistance improved in the aerobic and combined exercise groups but not in the resistance exercise group, compared with the control group. In addition, visceral fat decreased and endurance capacity improved in the aerobic and combined exercise group, whereas skeletal muscle mass and muscle strength were enhanced in the resistance and combined exercise group [33]. These results imply that combined exercise might be an optimal strategy for simultaneous improvement of visceral obesity and low muscle mass, which may in turn help ameliorate metabolic complications and functional limitations in the elderly.

There are several limitations to this study. First, the cross-sectional nature of this study did not allow us to identify causal relationships. Second, muscle quality (e.g., such as muscle strength and muscle fiber types) was not considered. In addition, due to relatively young study population, we used “low muscle mass” instead of the term “sarcopenia”. Finally, information on smoking status and alcohol consumption may be affected by recall bias since these data were self-reported. Despite these limitations, our results are strengthened by the use of gold-standard techniques to measure CRF (Vo_2max_ obtained through exercise on a cycle ergometer) and detect low muscle mass and visceral obesity (DXA and CT, simultaneously), and by measuring various markers of inflammation.

In conclusion, individuals with low CRF had an increased risk of having low muscle mass and combined low muscle mass and visceral obesity after adjusting for confounding factors, including insulin resistance and inflammation. This study may be a stimulant to provoke further research about the novel relationship between low CRF and having low muscle mass and combined low muscle mass and visceral obesity.
